# Can baseline serum creatinine and e-GFR predict renal function outcome after augmentation cystoplasty in children?

**DOI:** 10.1590/S1677-5538.IBJU.2017.0078

**Published:** 2018

**Authors:** Prempal Singh, Ankur Bansal, Virender Sekhon, Sandeep Nunia, M. S. Ansari

**Affiliations:** 1 Department of Urology and Renal Transplant, Sanjay Gandhi Postgraduate Institute of Medical Sciences, Lucknow, India

**Keywords:** Serum, Delayed Graft Function, Creatinine

## Abstract

**Objective:**

To assess cut-off value of creatinine and glomerular filtration rate for augmentation cystoplasty (AC) in paediatric age-group.

**Materials and Methods:**

Data of all paediatric-patients (<18 years) with small capacity bladder, in whom AC was advised between 2005-2015 were reviewed. All patients were divided in two-groups, AC-group and control-group (without AC). Creatinine and e-GFR were assessed at the time of surgery, at 6 months and at last follow-up. Renal function deterioration was defined as increase in creatinine by ≥25% from baseline value or new-onset stage-3 CKD or worsening of CKD stage with pre-operative-CKD stage-3. ROCs were plotted using creatinine and e-GFR for AC.

**Results:**

A total of 94 patients with mean-age 8.9 years were included. The mean creatinine and e-GFR were 1.33mg/dL and 57.68mL/min respectively. Out of 94 patients, AC was performed in 45 patients and in the remaining 49 patients AC was not done (control-group), as they were not willing for the same. Baseline patient's characteristics were comparable in both Groups. 22 underwent gastro-cystoplasty (GC) and 25 underwent ileo-cystoplasty (IC). Decline in renal function was observed in 15 (33.3%) patients of AC-group and in 31 (63.3%) patients of control-group. Patients having creatinine ≥1.54mg/dL (P=0.004, sensitivity (S) 63.6% and specificity (s) 90.5%) at baseline and e-GFR ≤46mL/min (P=0.000, S=100% and s=85.7%) at the time of surgery had significantly increased probability of renal function deterioration on follow-up after AC.

**Conclusion:**

e-GFR ≤46mL/min and creatinine ≥1.54mg/dL at time of surgery could serve as a predictor of renal function deterioration in AC in paediatric patients.

## INTRODUCTION

Augmentation cystoplasty [AC] is one of the major reconstructive surgeries performed in paediatric patients. Augmentation cystoplasty including gastro-cystoplasty and ileo-cystoplasty preserves renal function and provides urinary continence in most children with intractable lower urinary tract disease. Use of augmentation cystoplasty has decreased in developed countries like UK and USA in recent years (1, 2), however in many developing countries it is still one of the commonly performed surgeries in children. It may be due to relatively high prevalence of tuberculosis/parasitic infection and neglected congenital malformation in developing countries. Indications for augmentation cystoplasty include bladder capacity ≤65% of age, end filling pressures >40cm of water, intractable irritative lower urinary tract symptoms in spite of anticholinergics. Despite successful augmentation cystoplasty, many of these children progress to chronic kidney disease (CKD).

The reported long term outcomes following AC vary widely among different studies including decline in renal function, pyelonephritis, spontaneous bladder perforation, bladder calculi, and increased risk of malignancy following AC (3-7). Despite successful surgery, some of these children continue to have fall in renal function. Only few studies have reported on decline in renal function after augmentation cystoplasty, with higher incidence in lower urinary tract obstruction (8). However, none of the studies documented the association of baseline renal function and development of subsequent CKD after augmentation cystoplasty.

## MATERIALS AND METHODS

Data of all paediatric patients with small capacity bladder, in whom augmentation cystoplasty (AC) was advised between January 2005 to December 2015, were reviewed. All patients were divided in two Groups, augmentation cystoplasty Group (AC Group) who underwent AC and control Goup, in whom augmentation of bladder was not done. Glomerular filtration rate (e-GFR) was calculated using the Schwartz equation. Serum creatinine and e-GFR were assessed in AC Group at the time of surgery, then at 6 months and at last follow-up and in control Group at baseline, then at 6 months and at last follow-up. Renal function deterioration was defined as increase in serum creatinine by 25% or more than the base line value or new-onset stage-3 chronic kidney disease (CKD) or worsening of CKD stage with pre-operative CKD stage-3. Receiver Operator Curves (ROC) were plotted using serum creatinine and e-GFR for augmentation cystoplasty.

## RESULTS

94 patients with mean age 8.9 years (range 6 to 17 years) were included. Among 94 patients, 56 were boys and 38 were girls. The mean serum creatinine and e-GFR were 1.33 mg/dL (range 0.4 to 2.4mg/dL) and 57.68mL/min (range 28 to 82mL/min) respectively. Out of 94 patients, augmentation cystoplasty was performed in 45 patients (AC Group) and in the remaining 49 patients augmentation cystoplasty was not done (Control Group), as they were not willing for the same. Baseline patient's characteristics were comparable in both the groups (Table-1).

**Table 1:** Patient Characteristics.

Patients Characteristics		Augmentation Cystoplasty	Control group	P value
No of Patients		45	49	
Mean Age (Range)		9.34 (5-17)	8.57 (6-16)	0.8
**Gender**				
	Boys	27	29	1.0
	Girl	18	20	
**Diagnosis**				
	PUV	19	23	
	Neurogenic Bladder	08	20	
	Extrophy Bladder	07	00	0.68
	Epispadias	06	04	
	GUTB	05	02	
	Mean Baseline e-GFR (mL/min.)	58.73±21.8	56.72±19.5	0.63
**Baseline renal function**				
	Stage 0-II	20	22	1.0
	Stage III-V	25	27	
	Mean Baseline Serum Creatinine (mg/dL)	1.32±0.8	1.34±1.1	0.92
**Renal function outcome (N,%)**				
	Decline	15 (33.3%)	31 (63.3%)	0.004
	Stable	30 (66.7%)	18 (36.7%)	
	Mean e-GFR at last follow-up (mL/min.)	55.72±18.4	42.8±16.5	0.001
	Mean Serum Creatinine at last follow-up (mg/dL)	1.34±0.7	1.69±0.8	0.026

In AC Group, ileo-cystoplasty (IC) was done in 25 patients and 20 patients underwent gastro-cystoplasty (GC) as per patient's characteristics (Tables 2 and 3). Gastro-cystoplasty was performed in two-third male patients because of chronic kidney disease due to posterior urethral valve (PUV). Ileo-cystoplasty was performed in all thirteen patients with exstrophy/epispadias reconstruction. No posterior urethral valve patient underwent IC due to raised serum creatinine at the time of augmentation cystoplasty. Baseline mean serum creatinine was 1.30mg/dL and 1.35mg/dL in IC and GC Group respectively, while baseline mean e-GFR was 74mL/min and 39.75mL/min. Out of 94 patients, 52 had baseline chronic kidney disease (CKD) [46 patients had chronic kidney disease stage III and 6 had chronic kidney disease stage IV]. Among these 52 patients with baseline CKD, 25 patients were in AC Group and remaining 27 patients were in control Group. Gastro-cystoplasty was performed in 18 patients with chronic kidney disease (15 were in CKD stage III and all CKD stage IV) and ileo-cystoplasty was performed in 7 patients with CKD stage III.

**Table 2:** Baseline Renal Function and Underlying Pathology (AC group/Control group).

Underlying	No Of Patients	CKD Stage	CKD Stage	CKD Stage	CKD Stage	CKD Stage	CKD Stage
Pathology		0	I	II	III	IV	V
PUV (Total)	42						
AC group	19	-	-	05	12	02	-
Control group	23	-	-	11	10	02	-
Neurogenic Bladder (Total)	28						
AC group	08	-	01	01	05	01	-
Control group	20	01	01	05	12	01	-
Extrophy Bladder (Total)	07						
AC group	07	05	-	02	-	-	-
Control group	-	-	-	-	-	-	-
Epispadia (Total)	10						
AC group	06	04	02	-	-	-	-
Control group	04	04	-	-	-	-	-
GUTB (Total)	07						
AC group	05	-	-	-	05	-	-
Control group	02	-	-	-	02	-	-

**Table 3:** Baseline CKD stage Distribution In Ileo-cystoplasty And Gastro-cystoplasty.

Augmentation Cystoplasty	No of Patients	0	I	II	III	IV	V
Ileocystoplasty	25	12	02	05	07	-	-
Gastrocystoplasty	20	-	-	02	15	03	-

Decline in renal function was observed in 15 (33.3%) patients of AC Group and in 31 (61.3%) patients of control Group, which was significantly higher in control Group (p value: 0.004). Out of 15 patients with declined renal function in AC Group, 12 (60%) patients were in GC Group and 3 (12%) patients were in IC Group, maybe because 90% of GC Group patients had baseline CKD. Among these 12 patients in GC Group, 9 patients had baseline CKD stage III B and 3 had baseline CKD stage IV. Three patients who had deterioration of renal function in IC Group, had baseline CKD III B stage. The mean baseline e-GFR were 40 and 29.22mL/min in IC and GC Group respectively in patients, who had deterioration in renal function after augmentation cystoplasty. However, stable renal function was observed after 5.7 years of mean follow-up in 30 (66.7%) patients of AC Group and in 18 (36.7%) patients of control Group, which was significantly lower in control Group (p value: 0.004).

Mean e-GFR at last follow-up in AC and control Group was 55.7mL/min and 42.8mL/min respectively, which was significantly lower in control Group (p value: 0.001).

**Figure 1:**
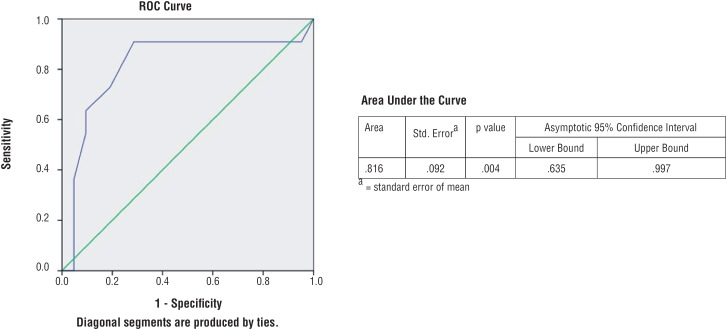
Receiver Operator Curve of Baseline Serum Creatinine for Augmentation Cystoplasty.

**Figure 2:**
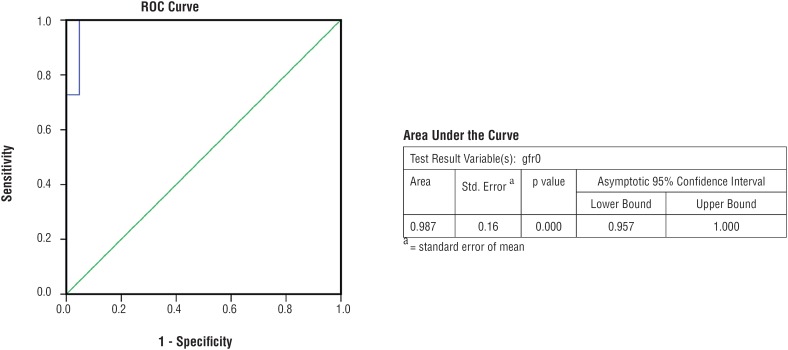
Receiver Operator Curve of Baseline e-GFR For Augmentation Cystoplasty.

Similarly, mean serum creatinine at last follow-up in AC and control Group was 1.34mg/dL and 1.69mg/dL, which was also significantly higher in control Group (p value: 0.026). Receiver Operator Curves (ROC) were plotted using serum creatinine and e-GFR. Patients having serum creatinine ≥1.54mg/dL (Figure-1) and e-GFR ≤4mL/min (Figure-2) at time of surgery had significantly increased probability of renal function deterioration with sensitivity 63.6% and specificity 90.5% (p=0.004) and sensitivity 100% and specificity 85.7% (p=0.00) respectively on follow-up after AC.

## DISCUSSION

The major goals of augmentation cystoplasty are to provide a compliant bladder with low end filling pressure, increase bladder capacity and or control bladder over activity. AC should allow the upper tracts to remain intact while preserving renal function and maintain urinary continence (9-13) In spite of a worldwide decrease, AC remains an option, with high patient acceptance rates, in both neurogenic and non-neurogenic bladder dysfunction when conservative treatments such as pharmacological methods and minimally invasive treatments like intravesical botulinum have been unsuccessful (14, 15).

In various studies the renal function has been reported to improve, remain stable or even worsen after AC (8, 9-11, 16). In spite of successful surgery, in some of the children the renal function continues to deteriorate. Most of the studies have described the metabolic complications, only few studies have reported on decline in renal function after AC (17) Further, none of the study documented the association of baseline renal function and development of subsequent CKD after augmentation cystoplasty.

In studies that have shown the fall in renal function, the rate of decline appeared to be related to the pre-operative creatinine clearance. Kuss et al., found deterioration in only 4.1% of patients who had a creatinine clearance of ≥40mL/min (17). In the present study, no patient required dialysis in immediate postoperative period. By 12 months of follow-up, the average increase in serum creatinine was 0.20mg/dL. It is unclear what was the cause of rise in creatinine in these patients. Possible etiologies could be absorption of creatinine from the intestinal mucosa or inherent progression of baseline chronic kidney disease. These require future studies using more objective measures. Even the use of stomach did not appear to be protective for further decline in renal function after GC, if the baseline e-GFR was less than 30 or CKD stage 4. However, mean baseline e-GFR for GC was 39.75mL/min, which means most of the patients who underwent GC had CKD at the time surgery and the reasons for post-GC decline in renal function might be further progression of baseline CKD or renal dysplasia (most being the cases of PUV) (18).

Overall, renal function deterioration was observed in 15 patients (33.3%) of AC Group at mean follow-up of 5.7 years. E. Fontaine et al. showed that lower urinary tract reconstruction was associated with a significant deterioration in renal function in 19% of patients after 10 years of follow-up (16); this incidence is much lower as compared to the present study, probably on account of the different patient population as they included only exstrophy patients. Congenital renal damage is rare in exstrophy patients before surgical correction, so that renal dysplasia or intrauterine nephropathy can be eliminated as a cause of any subsequent deterioration in renal function (18). In our study, 20 (44.4%) patients of AC Group and 25 (51.0%) patients of control group had baseline CKD due to lower urinary tract obstruction (LUTO).

Bruce J. Schlomer et al. also demonstrated that principal diagnosis was strongly associated with risk of decline in renal function or CKD after AC. A diagnosis of LUTO including posterior urethral valves (PUV) or neurogenic bladder was strongly associated with subsequent renal function deterioration (HR 13.7, 95% CI 9.7e19.9) (8). Developing CKD is likely related to primary pathology i.e. LUTO and not caused by augmentation cystoplasty itself (8).

Mean e-GFR of IC Group was 74mL/min and 7 patients had CKD stage III. Out of 07 patients, 03 (12%) patients had further progression of chronic kidney disease and both patients had e-GFR lower than 45mL/min and no hydroureteronephrosis (HDUN) or obstructive uropathy on ultrasonography. Among 20 patients of GC Group, 18 had CKD at the time of surgery with mean e-GFR 39.37mL/min. Fifteen patients were in CKD stage III and 3 were in CKD stage IV. Twelve (60%) patients, who underwent GC showed decline in renal function. All patients with CKD stage IV (n=3) and 9 patients with CKD stage III B in GC Group, noticed deterioration in renal function within one year after AC with baseline mean e-GFR 29.2mL/min. In the present study, it was found that the IC is a safe alternative for the patient who have e-GFR 46mL/min or more. For patients with baseline CKD stage-IIIB, GC is a worthwhile option up to e-GFR 38mL/min or more. Further, renal functional outcome in children after AC are poor, if e-GFR is 46mL/min or lower. Mean baseline serum creatinine in our study was 1.35mg% in patients who recorded decline in renal function after AC. Patients who maintained stable renal function after AC had a mean baseline serum creatinine of 1.2mg%. Authors acknowledge that this was a retrospective study and serum creatinine is influenced by patient age, sex and lean body mass. Intestinal segments used for augmentation cystoplasty might absorb creatinine from urine, which may result in falsely elevated serum creatinine measurements. More accurate measurement of renal function like cystatin c assessment would be helpful but was unavailable for this retrospective study. Besides, the study includes heterogeneous patients with wide range of diagnosis and baseline renal function. About 55.55% of patients had baseline chronic kidney disease (CKD stage III and stage IV), who underwent augmentation-cystoplasty. Ninety percent patients with baseline chronic kidney disease underwent GC in an attempt to decrease metabolic complications and stable renal function.

## CONCLUSIONS

Baseline GFR and serum creatinine both can predict the long-term renal outcome. e Glomerular filtration rate ≤4mL/min and serum creatinine 1.54mg/dL at time of surgery could serve as predictors of renal function deterioration in augmentation cystoplasty in paediatric patients.

## Abbreviations

AC: = Augmentation cystoplasty
IC: = Ileo-cystoplasty
GC: = Gastro-cystoplasty
LUTO: = Lower urinary tract obstruction
e-GFR: = Estimated Glomerular filtration rate (calculated using the Schwartz equation)
CKD: = Chronic kidney disease
ROC: = Receiver operator characteristic curve
